# Therapeutic Use of Extracellular Vesicles for Acute and Chronic Lung Disease

**DOI:** 10.3390/ijms21072318

**Published:** 2020-03-27

**Authors:** Erin N. Worthington, James S. Hagood

**Affiliations:** Division of Pulmonology, Department of Pediatrics, University of North Carolina at Chapel Hill, Chapel Hill, NC 27599, USA; nikki@unc.edu

**Keywords:** extracellular vesicles, lung disease, mesenchymal stem cells, pulmonary disease

## Abstract

Multipotent mesenchymal stem cells (MSCs) possess regenerative properties and have been shown to improve outcomes and survival in acute and chronic lung diseases, but there have been some safety concerns raised related to MSC-based therapy. Subsequent studies have demonstrated that many of the regenerative effects of MSCs can be attributed to the MSC-derived secretome, which contains soluble factors and extracellular vesicles (EVs). MSC-derived extracellular vesicles (MSC-derived EVs) replicate many of the beneficial effects of MSCs and contain a variety of bioactive factors that are transferred to recipient cells, mediating downstream signaling. MSC-derived EV therapy holds promise as a safe and effective treatment for pulmonary disease, but there remain many scientific and clinical questions that will need to be addressed before EVs are widely applied as a therapy. To date, the use of MSC-derived EVs as a treatment for lung disease has been conducted primarily in in vitro or pre-clinical animal models. In this review, we will discuss the current published research investigating the use of EVs as a potential therapeutic for acute lung injury/acute respiratory distress syndrome (ALI/ARDS), bronchopulmonary dysplasia (BPD), idiopathic pulmonary fibrosis (IPF), pulmonary arterial hypertension (PAH), asthma, and silicosis.

## 1. Introduction

Stem cell research has garnered significant attention over the last few decades, especially regarding potential applications for the regeneration of damaged or diseased tissue [1]. Stem cells are self-renewing and undifferentiated cell types that are classified by their potential to differentiate into functional cells and further described as totipotent, pluripotent, multipotent, and unipotent [1,2,3]. Multipotent stem cells are able to differentiate into cell types of a particular cellular lineage. Mesenchymal stem cells (MSCs) are multipotent cells found in multiple anatomic compartments (e.g., bone marrow, adipose tissue, umbilical cord, and lungs) which, in addition to serving as progenitors for connective tissue cells, are able to stimulate the growth, repair, and survival of other cells and tissues [4]. MSCs have been shown to have a beneficial immunomodulatory and regenerative capacity. There is a significant body of research published describing MSCs as a potential therapy for several acute and chronic lung diseases, summarized in recently published comprehensive reviews [3,5,6]. While many experimental and clinical studies have established the use of MSCs in lung disease, there have been some safety concerns raised related to MSC-based therapy, which include the undesirable differentiation of transplanted MSCs resulting in possible malignant transformation, as well as vascular occlusion caused by injected MSCs [7]. More recent studies have discovered that the MSC secretome contains soluble factors and extracellular vesicles (EVs), which can mimic many of the desired clinical effects of MSCs [8]. EVs are hypothesized to be a safer alternative to MSCs since they are cell free and appear to have a better immunogenicity, tumorigenicity, and embolism formation side effect profile than MSCs [9,10]. This has led to increased efforts to develop MSC-derived extracellular vesicles (MSC-derived EVs) as a potential therapeutic agent.

## 2. Extracellular Vesicles

EVs are naturally occurring, cell-derived membrane-bound spherical structures that are shed or secreted from most cell types under various physiologic and pathologic conditions into the circulation or surrounding body fluids. EVs have been found in a variety of biological fluids, including blood, bile, bronchoalveolar lavage fluid (BALF), and urine [11]. EVs are important for cell-to-cell communication as they provide a way for cells to exchange cellular components, which can influence a broad range of signaling pathways [12].

EVs range between 30–5000 nm in diameter, and until recently have been classified based on size, which included exosomes (30–100 nm), microvesicles/ectosomes (100–1000 nm), and apoptotic bodies (100–5000 nm). These categories of EVs also have distinct mechanisms of release from cells, as described below. The International Society for Extracellular Vesicles (ISEV) now recommends using “extracellular vesicle” as an umbrella term to describe vesicles of all sizes, due to the size heterogeneity and lack of specific markers for each EV size classification [13]. The most recent ISEV guidelines recommend EVs be described by physical characteristics, biochemical composition, or descriptions of conditions or cells of origin in place of the former size-based terms [13]. Furthermore, additional guidelines in this position statement describe the minimal experimental requirements needed when performing experiments with EVs [13].

The formation of EVs include three distinct biogenetic pathways. First, exosome biogenesis is dependent on the endosomal sorting complexes required for transport (ESCRT) protein for the formation of endosomal intraluminal vesicles resulting in multivesicular bodies (MVBs), from which exosomes are released via exocytosis when MVBs fuse with the plasma membrane [14,15]. This pathway has been exploited by viruses for viral egress from an infected cell [16]. Second, microvesicles are shed via the outward blebbing of the plasma membrane and then released via proteolytic cleavage [14]. Third, apoptotic bodies are formed by membrane blebbing during apoptotic cell disassembly.

EVs express cell surface proteins similar to the cells from which they are derived and contain various cytoplasmic components, including proteins, RNA (including mRNA and noncoding RNA such as microRNA (miRNA)), lipids, mitochondria, and DNA [12]. The contents of EVs reflect their modes of biogenesis, cellular origin, physiological or culture conditions, and exogenous stimuli. It has been shown that exposure of MSCs to hypoxia or growth factors increased EV production and altered EV cargo, resulting in enhanced effects [17,18,19]. The determinants of EV contents are not well understood and may include both passive and regulated mechanisms. Overall, the individual function of an EV depends not just on its contents but also on the expression of biomarkers on its plasma surface. Plasma-membrane-derived EVs may contain putative markers: TyA, C1q, Arrestin domain-containing protein 1 (ARRDC1), and CD73. Endosome-derived EVs may express distinct biomarkers, including tetraspanins (CD61, CD63, CD81), ESCRT proteins (TSG101 and Alix), syntenin, flotillin, and heat shock proteins. These biomarkers may suggest a biogenetic lineage but are not always specific as there can be some cross-over among these.

The cellular targeting, uptake, and plasma membrane fusion of EVs is another area of ongoing investigation that is proving to be complex and multifaceted [12,15,20,21]. The interaction of EVs with recipient cells involves numerous molecular interactions that affect various downstream signaling events and can include signaling through membrane proteins, binding to cell surface receptors, and/or the delivery of the EV cargo (e.g., Protein, DNA, RNA, miRNA, and lipids) to various intracellular compartments via EV uptake via either cell membrane fusion or endocytosis. Thus, EVs are able to mediate intracellular communication via shuttling bioactive signaling molecules as cargo or by the direct activation of signaling pathways to a recipient cell.

## 3. MSC-Derived EVs in Models of Lung Disease

**Figure 1:**
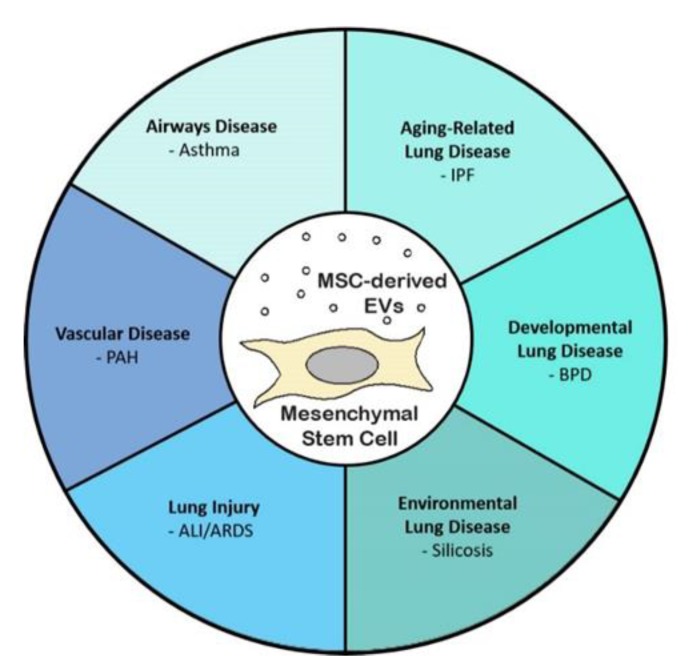
Multipotent mesenchymal stem cell-derived extracellular vesicles (MSC-derived EVs) as a potential therapeutic for lung diseases. The in vitro and pre-clinical studies evaluating the potential therapeutic role of MSC-derived EVs have shown promising results. in many different categories of lung diseases.

The use of MSC-derived EVs as a potential therapy for lung disease is a fairly young but rapidly growing field, with current research covering a wide variety of lung diseases (Figure 1) [22]. The majority of the current research evaluating the therapeutic potential of EVs has been performed in in vitro or pre-clinical animal model systems (Table 1). This review will describe current published research using EVs as a potential therapy for acute lung injury/acute respiratory distress syndrome (ALI/ARDS), bronchopulmonary dysplasia (BPD), idiopathic pulmonary fibrosis (IPF), pulmonary arterial hypertension (PAH), asthma, and silicosis.

### 3.1. Acute Lung Injury/Acute Respiratory Distress Syndrome

Acute lung injury and acute respiratory distress syndrome (ALI/ARDS) is a disease that carries a high burden of morbidity and mortality [23,24,25]. It is characterized by rapid onset respiratory failure resulting from various direct and indirect insults to the lung parenchyma or vasculature. The pathophysiology includes a severe inflammatory response and high levels of circulating cytokines resulting in ongoing damage to lung tissue. Treatment with anti-inflammatories has been ineffective at reducing mortality rates or improving outcomes in patients with ALI/ARDS [26]. Several ALI/ARDS model systems have shown treatment with MSC-derived EVs resulted in a reduction in lung injury severity.

MSC-derived EVs have demonstrated beneficial effects in both bacterial- and viral-induced ALI. In an influenza-induced ALI porcine model, intratracheal (IT) treatment with swine bone marrow MSC-derived EVs 12 h after influenza virus infection resulted in reduced viral replication, viral shedding, and reduced production of pro-inflammatory cytokines [27]. In an *Escherichia coli* pneumonia murine model, Monsel and colleagues demonstrated that the intravenous (IV) administration of human bone marrow MSC-derived EVs improved survival and reduced inflammation [28]. This study also shows that MSC-derived EVs were effective in vitro via increased human macrophage bacterial phagocytosis, reduced inflammation, and increased ATP levels in human alveolar type 2 cells. Another study showed human bone marrow MSC-derived EVs protected against ALI by promoting an anti-inflammatory and a highly phagocytic macrophage phenotype, and demonstrating a role for EV-mediated mitochondrial transfer in this observed effect [29].

A variety of studies have used an endotoxin-mediated in vivo model to evaluate the effects of MSC-derived EVs for ALI/ARDS. For instance, the IT instillation of human bone marrow MSC-derived EVs in a murine *E. coli* endotoxin-induced ALI model resulted in improvements in pulmonary edema and lung protein permeability due to reduced extravascular lung water and total protein in BALF [30]. Additionally, MSC-derived EV-treated ALI mice demonstrated a reduction in neutrophil infiltration and macrophage inflammatory protein-2 (MIP-2) levels in the alveoli, suggesting anti-inflammatory effects [30]. Furthermore, this study suggests a possible role of keratinocyte growth factor mRNA transfer and subsequent protein expression to the injured alveolar epithelium [30]. Tang and colleagues demonstrated that the IT instillation of human bone marrow MSC-derived EVs in a murine lipopolysaccharide (LPS)-induced ALI model resulted in reduced inflammation by a reduction in the influx of white blood cells, neutrophils, and MIP-2 secretion [31]. In addition, the MSC-derived EVs contained angiopoietin-1 (Ang-1) mRNA, and Ang-1 protein expression in alveolar cells was increased in the EV treated mice. The knockdown of Ang-1 in the MSC parent cells partially ameliorated the beneficial effects of the MSC-derived EVs, suggesting that the transfer of Ang-1 mRNA by EVs is important for the protective effects in this system. This study also showed that EVs suppressed the secretion of TNF-alpha and inflammatory cytokines, while increasing the secretion of an anti-inflammatory cytokine, interleukin-10 (IL-10), in a mouse macrophage cell line. Another study showed that MSC-derived EVs restored partial protein permeability across injured human lung microvascular endothelial cells by increasing the transfer of Ang-1 mRNA to the injured endothelium, with resulting increased expression and secretion by injured human lung microvascular endothelial cells [32]. This is significant because Ang-1 is an angiogenic factor that is involved in endothelial cell stabilization during injury, reducing leukocyte–endothelium interactions, and endothelium permeability. Wang and colleagues showed that MSC-derived EVs were able to stabilize the endothelial barrier function of pulmonary microvascular endothelial cells in vitro after treatment with LPS; the knockdown of hepatocyte growth factor inhibited this effect, suggesting that hepatocyte growth factor may have a role in the regulation of endothelial permeability by MSC-derived EVs [33]. Gennai et al. found that MSC-derived EVs increased alveolar fluid clearance in human donor lungs in an ex vivo ischemia/reperfusion-induced ALI model and the expression of CD44 on the MSC-derived EVs was necessary for EV uptake by the recipient cell and downstream effects [34].

### 3.2. Bronchopulmonary Dysplasia

Bronchopulmonary dysplasia (BPD), a leading cause of morbidity and mortality in premature infants, is a chronic lung disease that is multifactorial in origin [35]. The lungs of infants with BPD are underdeveloped due to the arrest or delay of alveolar development and pulmonary angiogenesis. These patients are at increased risk of pulmonary infection, inflammation, and injury throughout infancy and childhood. There is a significant body of research evaluating the utility of using MSCs as a possible therapy for BPD, as highlighted in several recent reviews [35,36,37]. However, given the success of MSC-derived EVs in other pulmonary diseases, there are now multiple studies looking at the role of MSC-derived EV as therapy for BPD. Willis and colleagues found that umbilical cord MSC-derived EVs improved lung function, decreased both fibrosis and pulmonary vascular remodeling, and resulted in an improvement in pulmonary hypertension in a neonatal murine hypoxia model [38]. EV treatment decreased hyperoxia-induced inflammation and altered the hyperoxic lung transcriptome. The EVs were taken up by alveolar macrophages and suppressed the macrophage M1-like inflammatory state and increased the M2-like anti-inflammatory state both in vitro and in vivo. Chaubey et al. demonstrated in a neonatal murine BPD model that umbilical cord MSC-derived EVs reduced pulmonary inflammation, pulmonary hypertension, and right ventricular hypertension partially mediated through the exosomal protein tumor necrosis factor alpha-stimulated gene-6 (TSG-6) [39]. In another report, umbilical cord MSC-derived EVs were as effective as the parental MSCs in protecting against hypoxia-induced lung injury in neonatal rats [40]. These beneficial effects were partially attenuated by knocking down the vascular endothelial growth factor (VEGF) gene in MSCs prior to EV isolation.

### 3.3. Idiopathic Pulmonary Fibrosis

Idiopathic Pulmonary Fibrosis (IPF) is a chronic and progressive fibrosing interstitial lung disease with pathophysiology characterized by fibroblast proliferation and extracellular matrix remodeling [53]. IPF has multiple genetic associations but no clearly defined cause and few effective treatments. Pirfenidone and nintedanib are the only currently United States Food and Drug Administration (FDA)-approved options for treatment, but they do not significantly ameliorate respiratory symptoms, improve the rate of acute exacerbations, or prevent the eventual decline of lung function [54,55,56,57]. Stem cell therapy has been used as a treatment for pulmonary fibrosis in pre-clinical animal models and in human patients with IPF. In animal models, these studies have demonstrated that adult stem cells can help to prevent the progression of pulmonary fibrosis in the bleomycin-induced pulmonary fibrosis model [58,59]. In human studies, MSC-based therapy has been shown to be safe and well-tolerated and improved quality-of-life parameters in phase 1 clinical trials [60,61,62]. As with other pulmonary diseases, there is now increasing interest in using the EVs released from mesenchymal stem cells for IPF therapies.

A study by Mansouri et al. demonstrated that intravenous human bone marrow MSC-derived EVs could prevent and reverse intratracheal bleomycin-induced pulmonary fibrosis in mice with resulting improvements in both pulmonary morphology and lung architecture and decreased collagen deposition [41]. They also showed the MSC-derived EVs were immunomodulatory and reduced monocyte-driven inflammation. Tan and colleagues demonstrated that intranasally-instilled human amnion epithelial-derived EVs, but not fibroblast-derived EVs, reduced lung inflammation and can both prevent or resolve bleomycin-induced lung injury depending on the timing of EV treatment after bleomycin injury [42]. The amnion epithelium-derived EVs also had a direct effect on immune cells, including increased macrophage phagocytosis, reduced neutrophil myeloperoxidase, and suppressed T cell proliferation. Shentu and colleagues used human bone marrow MSC-derived EVs to suppress the transforming growth factor beta 1 (TGFβ1)-stimulated myofibroblast differentiation of normal and IPF lung fibroblasts [63]. They found that MSC-derived EVs utilized a Thy1-integrin dependent pathway for cell-to-cell communication for uptake into fibroblasts and the delivery of EV cargo and resulting anti-fibrotic activity. The MSC-derived EVs contained several miRNAs, which target pro-fibrotic genes that are upregulated in IPF fibroblasts. In another study conducted by Shentu and colleagues, the IV injection of MSC-derived EVs into mice 14 days after bleomycin-induced pulmonary injury resulted in decreased pulmonary fibrosis. This model suggests a possible therapeutic effect of bone marrow MSC-derived EVs on established lung fibrosis by modulating the myofibroblastic phenotype [43].

### 3.4. Pulmonary Arterial Hypertension

Pulmonary Arterial Hypertension (PAH) refers to hemodynamic alterations of the pulmonary circulation in which the pulmonary artery pressures are >25 mm Hg. PAH is a progressive disease characterized by the remodeling of the pulmonary arteries, increased pulmonary infiltrates, loss of vascular cross-sectional area, and elevated pulmonary vascular resistance leading to right heart failure and death [64,65]. A key pathological feature of PAH is vascular remodeling, which includes the accumulation of various cells, including pulmonary artery smooth muscle cells, endothelial cells, fibroblasts, myofibroblasts, and pericytes, in the pulmonary arterial wall [66]. In addition, there is loss of pre-capillary arteries and perivascular infiltration of inflammatory cells [66]. Despite significant research directed at therapies for PAH, there are limited options available, and the current treatments do not improve the abnormal pulmonary vascular remodeling or inflammation seen in this disease. The goal in PAH is to find a therapy that would reverse the key features of PAH and result in the subsequent regeneration of normal pulmonary vessels. Thus, there is active research in exploring MSC-derived EVs as a possible PAH treatment.

To evaluate the effect of MSC-derived EVs on pulmonary vasculature, Lee and colleagues used a murine hypoxia-induced pulmonary hypertension murine model [44]. They demonstrated that IV treatment with MSC-derived EVs, but not fibroblast EVs, suppressed the influx of lung macrophages, the induction of multiple pro-inflammatory and proliferative mediators, and inhibited vascular remodeling, protecting against the development of right ventricular hypertrophy (RVH) and resultant PAH. The MSC-derived EV treatment decreased the signal transducer and activator of transcription 3 (STAT3)-mediated signaling induced by hypoxia, which is a key molecule involved in the response of pulmonary vasculature to hypoxia. They also found that MSC-derived EVs increased the lung expression levels of miR-204, an miRNA that is known to be suppressed in human pulmonary hypertension. Another group used a rat monocrotaline-induced PAH model and found that the IV injection of both MSCs and MSC-derived EVs resulted in a reduced mean pulmonary artery pressure and mean right ventricular pressure with a subsequent decrease in RVH [45]. This suggests that, in this system, the MSC-derived EVs are as effective as their cell of origin in preventing PAH. Using a similar monocrotaline-induced PAH model, Aliotta et al. demonstrated that bone marrow MSC-derived EVs could induce or prevent the development of PAH depending on the miRNA cargo [46]. In this study, IV treatment with EVs from monocrotaline injured mice induced RV hypertrophy and pulmonary vascular remodeling, whereas murine and human MSC-derived EVs reversed MCT-induced RV hypertrophy and pulmonary vascular remodeling. This group further separated out the MSC-derived EVs based on particle size and found that the MSC exosome fraction had a greater therapeutic effect compared to the microvesicle fraction. This difference was attributed, in part, to the MSC exosome fraction being enriched with anti-inflammatory and anti-proliferative miRNAs, including miRs-34a, -122, -124, and -127, compared to the exosomes harvested from the plasma of healthy mice. Another report using the Sugen/hypoxia pulmonary hypertension rat model, demonstrated that MSC-derived EVs could prevent and reverse pulmonary hypertension with a subsequent reduction in RVH and pulmonary vascular remodeling [47].

### 3.5. Silicosis

Silicosis is an occupational disease caused by the inhalation of dust containing crystalline silica particles, resulting in lung inflammation and leading to a progressive and irreversible lung fibrosis [67,68]. Choi and colleagues were the first to show that human bone marrow MSC-derived EVs were able to reduce the influx of inflammatory cells and collagen deposition in a murine silicosis pulmonary fibrosis model [50]. Phinney et al. reported that human bone marrow MSC-derived EVs used arrestin domain-containing protein 1-mediated microvesicles to target depolarized mitochondria to the plasma membrane [52]. These vesicles are then taken up by macrophages and result in enhanced bioenergetics. Furthermore, they showed that miRNA contained in the EVs could inhibit macrophage activation by suppressing Toll-like receptor (TLR) signaling to desensitize the macrophage to the ingested mitochondria. They also demonstrated that the IV injection of MSC-derived EVs in the lung of silica-exposed mice reduced lung inflammation and the fibrotic response. Bandeira and colleagues demonstrated that intratracheal installation of adipose MSC-derived EVs in a silicosis mouse model led to a reduction in collagen deposition, granuloma size, and macrophage influx [51]. Furthermore, they showed a reduction in the expression of Interleukin 1β and transforming growth factor β. Overall, the MSC-derived EVs reduced inflammation and fibrosis in a dose-dependent manner in this murine model of silicosis.

### 3.6. Asthma

Asthma is a chronic inflammatory lung disease characterized by intermittent airway obstruction, inflammation, increased mucus production, and bronchial hyperresponsiveness. There are multiple therapies on the market, but many patients continue to have poor disease control despite maximal treatment with current therapies. As a result, there is ongoing research to identify novel asthma therapies to both control and treat asthma exacerbations. For example, Cruz and colleagues successfully used MSC-derived EVs to alleviated allergic airway inflammation after the repeat mucosal exposures of *Aspergillus* hyphal extract in a murine model of severe asthma [48]. In another study, de Castro and colleagues used adipose-derived MSCs and MSC-derived EVs in an ovalbumin-induced allergic asthma murine model and found reduced inflammation and a reversal of tissue remodeling [49]. While the effects between MSCs and MSC-derived EVs were similar in this experiment, there were some differences in eosinophil counts and interleukin-4 (IL-4), interleukin-13 (IL-13), and eotaxin in lung tissue and CD3^+^CD4^+^ T cells in BALF and in static lung elastance. This data suggests that MSCs and MSC-derived EVs have similarities and differences in their downstream immunomodulatory effects. In a different study, MSC-derived EVs were able to promote regulatory T cell proliferation by increasing the expression of IL-10 and TGF-β1 in peripheral blood mononuclear cells from asthmatic patients [69].

## 4. Therapeutic Development of EVs for Lung Injury and Disease

MSC-derived EVs are now being considered as an effective and safer alternative to live MSC transplantation and have been shown to have therapeutic benefit in numerous pre-clinical models of pulmonary injury and disease. Nonetheless, the research is still in its infancy and there are still many unanswered questions that need to be addressed before MSC-derived EVs can become serious candidates as a clinical therapeutic.

There will need to be standardized protocols for the production, purification, characterization, and storage of EVs, as currently there are no universally accepted protocols. The techniques used for EV quantification include protein concentration, nanoparticle tracking analysis, tunable resistive pulse sensing, vesicle flow cytometry, surface plasma resonance, and electron microscopy. There are many recent reviews describing the predominant methods currently being used in the field [11,13,70,71,72]. The culture conditions and external stimuli of MSCs during EV production can alter the rate of EVs secreted and the EV composition, with resultant changes in the observed effects [17,18,19,73]. For example, MSCs pretreated with interleukin 1 beta (IL-1β) had an increased ability to induce the polarization of macrophages towards an anti-inflammatory M2 phenotype. This effect was due to paracrine activity modulated via the upregulation and transfer of the exosomal miR-146 [18]. Another study found that EVs from MSCs pretreated with LPS also enhance the polarization of macrophages towards an anti-inflammatory M2 phenotype [19]. The mechanisms behind how these external factors alter the EV cargo and effects is unknown. Understanding how the EV contents are altered in response to stimuli and what downstream effects those alterations have will be important to both the basic mechanistic understanding of how EVs signal. This may allow for exploitation of this pathway to increase the production of EVs and enhance their function.

MSCs are harvested from a variety of donors, hence a better understanding of how donor characteristics may alter EV function will also need to be explored. There is much interest in personalizing medicine, and MSC-derived therapies are ideally suited for this approach, as autologous MSCs could be used to generate personalized EVs as a therapy. Thus, fully understanding the donor effects on EVs would become very important when considering using autologous EVs as therapy.

The treatment of lung diseases will require EV administration via IT instillation or IV injection. The optimal route of administering EVs and the bio-distribution of EVs based on the cellular origin of the EV and the route of administration will need to be clarified. Currently, there are no published reports of the bio-distribution of EVs after IT instillation. However, the intravenous injection of EVs into mice has been shown to initially localize primarily to the spleen and liver and then rapidly redistribute to the gastrointestinal tract and lungs, followed by renal and hepatic clearance [74,75]. There also needs to be a better understanding of dose-response studies with EVs to establish EV dosing concentration, half-life, and whether repeat dosing will be necessary to reach optimal plasma or tissue levels. As with many therapeutics, chronic dosing is necessary so the effectiveness over time and the potential for a possible drug tolerance to form will also need to be explored. Moreover, in order to scale-up EV production to meet the levels needed for clinical uses, there will need to be well-defined methods and protocols that maintain a homogenous EV consistency and purity. In addition, understanding the ideal EV storage conditions which maintain stability and efficacy will have to be developed. Thus, there are many biological and technical hurdles that have to be addressed in order for MSC-derived EVs to be developed as a therapeutic.

## 5. Conclusions

In conclusion, we have summarized the current literature on the use of MSC-derived EVs as a treatment for acute and chronic lung disease. MSCs have shown promise for their regenerative and immune modulating effects but have been questioned as a therapeutic due to possible deleterious side effects. MSC-derived EVs are shown to be able to mediate many of the beneficial effects of MSCs, and it is postulated that they have fewer negative side-effects. Pre-clinical animal models have demonstrated that MSC-derived EV-based therapy may be a viable therapeutic option for the reversal or prevention of various lung diseases. This beneficial effect appears to be the result of the activation of the signaling pathways via the transfer of EV contents containing miRNA, RNA, and proteins; however, the molecular mechanisms of the different EV components is poorly understood. The current literature using MSC-derived EVs as a treatment for lung diseases in pre-clinical animal models use different methods of quantifying the dose of EVs, making direct comparison between the studies impossible. A consensus on the best method to define the EV dose needs to be determined so the therapeutic effectiveness of EVs can be compared across studies. In addition, there remains the challenge of scaling-up the production of EVs to a level that could be produced with clinical-grade quality and quantity. Thus, there are many questions and technical issues that need to be addressed before MSC-derived EVs can make the transition from animal models to humans. If these different challenges can be overcome, then MSC-derived EVs hold great promise as a potential therapeutic for numerous lung diseases.

## Figures and Tables

**Table 1:** EV treatment in preclinical models of lung disease.

	EV Origin	Model System		EV			Outcomes	Key Modulator	Ref.
		Species	Injury	Route	Dose	Isolation Method			
**ALI/ARDS**	Swine BM-MSCs	pig	Influenza	IT	EVs released by 10 × 10^6^ MSCs over 48 h	UCF	• ↓ Virus replication and shedding and inflammatory cytokines	EV RNA transfer to epithelial cells	[27]
	Human BM-MSCs	mouse	*E. coli* pneumonia	IT/IV	EVs released by 3–6 × 10^6^ MSCs (IT) or 9 × 10^6^ MSCs (IV) over 48 h	UCF	• ↑ Survival	EV transfer of KGF mRNA	[28]
							• ↓ Influx of inflammatory cells, cytokines, and bacterial load		
	Human BM-MSCs	mouse	LPS	Ex vivo	EVs released by 1.5 × 10^7^ MSCs over 48 h	UCF	• ↓ Lung inflammation and injury	EV-mediated mitochondrial transfer	[29]
							• Promoted anti-inflammatory and phagocytic macrophage phenotype		
	Human BM-MSCs	mouse	*E. coli* endotoxin	IT/IV	EVs released by 3 × 10^6^ MSCs over 48 h	UCF	• ↓ Pulmonary edema, lung protein permeability, influx of neutrophils, and MIP-2 expression	KGF-expressing EV transfer	[30]
	Human BM-MSCs	mouse	LPS	IT	EVs released by 3 × 10^6^ MSCs over 48 h	UCF	• ↓Lung inflammation, influx of neutrophils, and MIP-2 secretion	EV transfer of Ang-1 mRNA	[31]
							• Restored the pulmonary capillary permeability		
**BPD**	Human UC-MSCs and BM-MSCs	mouse	Hyperoxia-newborn	IV	EVs released by 0.5 × 10^6^ MSCs over 48 h	UCF (Optiprep)	• ↓ PAH, alveolar simplification, lung fibrosis, and vascular remodeling	EV mediated macrophage modulation	[38]
							• Improved PFTs		
							• ↑ Anti-inflammatory macrophage phenotype		
	Human UC-MSCs	mouse	Hyperoxia-newborn	IP	2.5 μg protein	UCF	• ↓ Lung inflammation, alveolar simplification, PAH, and RVH	Effects partially modulated via TSG-6	[39]
							• ↓ Cell death in brain and hypo-myelination		
	Human UC-MSCs	rat	HIE newborn	IT	6 × 10^9^ EVs	Tangential flow filtration	• ↓ Thickness of small pulmonary vessels and alveolar simplification		[40]
**IPF**	Human BM-MSCs	mouse	Bleomycin lung fibrosis	IV	EVs released by 5 × 10^6^ MSCs over 48 h	UCF (Optiprep)	• ↓ Lung fibrosis, lung inflammation, and inflammatory phenotype of monocytes and macrophages		[41]
	Human amnion epithelial cells	mouse	Bleomycin lung fibrosis	IV/IN	10 μg protein	UCF	• ↓ Lung fibrosis and inflammation		[42]
							• ↑ Macrophage phagocytosis and suppressed T cell proliferation		
	Human BM- MSCs	mouse	Bleomycin lung fibrosis	IV	50 μg protein	UCF	• ↓ Lung fibrosis	Thy-1 EV expression causes↑ EV effects	[43]
**PAH**	Mouse BM-MSCs and Human UC-MSCs	mouse	Hypoxic PAH	IV	10 μg protein	UCF	• ↓ Lung inflammation and pulmonary influx of macrophages	Inhibition of STAT3 signaling and ↑ of EV miR-17 superfamily of microRNA	[44]
	Rat BM-MSCs	rat	MCT PAH	IV	30 μg protein	UCF	• ↓ PAH, mean PA pressure, mean RV pressure, and RVH		[45]
	Murine and Human BM-MSCs	mouse	MCT PAH	IV	25 μg protein	UCF	• ↓ PAH, and RVH and vascular remodeling	↑ EV miRNAs that ↑ anti-proliferative, apoptotic, or senescent effects	[46]
	Human BM-MSCs	rat	Sugen/hypoxia PAH	IV	100 µg protein/kg	UCF	• ↓ RV pressure, RVH, muscularization of peripheral pulmonary vessels, and lung macrophages		[47]
**Asthma**	Mouse and Human BM-MSCs	mouse	Aspergillus extract hyphae	IV	EVs released by 3 × 10^6^ MSCs over 48 h	UCF	• ↓ Airway hyperreactivity and Th2/Th17-mediated airway inflammation		[48]
	Human AD-MSCs	mouse	Ovalbumin	IV	37 μg protein	UCF	• ↓ Lung elastance, collagen deposition, lung and BALF eosinophils, and BALF T lymphocytes		[49]
							• ↑ Treg cells in BALF and modulation of lung cytokines		
**Silicosis**	Human BM-MSCs	mouse	Silica-induced fibrosis	IV	10 μg protein	ExoQuick	• ↓ Lung fibrosis, inflammatory cells in airways and collagen deposition in lung parenchymal		[50]
	Mouse AD-MSCs	mouse	Silica-induced fibrosis	IT	EVs released by 1 × 10^6^ MSCs over 24 h	UCF	• ↓ Lung collagen content, granuloma size, and number of macrophages inside granuloma and in the alveolar septa		[51]
							• ↓ Expression inflammatory cytokines and lung static elastance		
	Human BM-MSCs	mouse	Silica-induced fibrosis	IV	40 μg protein	UCF	• ↓ Lung nodules, WBC influx, and levels of inflammatory and pro-fibrotic genes and enhanced macrophage energetics	Effects partially modulated EV transfer of mitochondria and miRNAs	[52]
							• Inhibition of TLR signaling in macrophages		

AD: Adipose tissue, ALI: Acute lung injury, Ang-1: Angiopoietin-1, ARDS: Acute respiratory distress syndrome, BALF: Bronchoalveolar lavage fluid, BM: Bone Marrow, BPD: Bronchopulmonary dysplasia, EV: Extracellular Vesicle, HGF: Hepatocyte Growth Factor, HIE: Hypoxic ischemic encephalopathy, IN: Intranasal, IP: Intraperitoneal, IT: Intratracheal, IV: Intravenous, KGF: keratinocyte growth factor, LPS: Lipopolysaccharide, miRNA: microRNA, MIP-2: Macrophage Inflammatory Protein *2*, MCT: Monocrotaline, MDMs: monocyte-derived macrophages, MSCs: Mesenchymal stem cells, PA: Pulmonary Artery, PAH: Pulmonary arterial hypertension, PFTs: pulmonary function tests, RV: Right ventricular, RVH: Right ventricular hypertrophy, Thy-1: Thymocyte differentiation antigen 1, TSG-6: tumor necrosis factor alpha-stimulated gene-6, UC: Umbilical cord, UCF: Ultracentrifugation, WBC: White blood cells.

## Abbreviations

AD	Adipose tissue
ALI	Acute lung injury
Ang-1	Angiopoietin-1
ARDS	Acute respiratory distress syndrome
BM	Bone Marrow
BALF	Bronchoalveolar lavage fluid
BPD	Bronchopulmonary dysplasia
ESCRT	Endosomal sorting complexes required for transport
EV	Extracellular Vesicle
HGF	Hepatocyte Growth Factor
HIE	Hypoxic ischemic encephalopathy
IL	Interleukin
IN	Intranasal
IP	Intraperitoneal
IT	Intratracheal
IV	Intravenous
KGF	Keratinocyte growth factor
LPS	Lipopolysaccharide
miRNA	microRNA
MIP-2	Macrophage inflammatory protein *2*
MDMs	Monocyte-derived macrophages
MSCs	Mesenchymal stem cells
MSC-derived EVs	Mesenchymal stem cell-derived extracellular vesicles
MVBs	Multivesicular bodies
PAH	Pulmonary arterial hypertension
Thy1	Thymocyte differentiation antigen 1
TGFβ1	Transforming growth factor beta 1
TSG-6	Tumor necrosis factor alpha-stimulated gene-6
UC	Umbilical cord
WBC	White blood cell
